# The role of cytidine 5′‐triphosphate synthetase 1 in metabolic rewiring during epithelial‐to‐mesenchymal transition in non‐small‐cell lung cancer

**DOI:** 10.1002/2211-5463.13860

**Published:** 2024-07-19

**Authors:** Fumie Nakasuka, Akiyoshi Hirayama, Hideki Makinoshima, Seiji Yano, Tomoyoshi Soga, Sho Tabata

**Affiliations:** ^1^ Institute for Advanced Biosciences Keio University Tsuruoka Japan; ^2^ Systems Biology Program, Graduate School of Media and Governance Keio University Fujisawa Japan; ^3^ Department of Molecular Pathology, Graduate School of Medicine The University of Tokyo Japan; ^4^ Tsuruoka Metabolomics Laboratory National Cancer Center Tsuruoka Japan; ^5^ Shonai Regional Industry Promotion Center Tsuruoka Japan; ^6^ Division of Translational Informatics, Exploratory Oncology Research and Clinical Trial Center National Cancer Center Kashiwa Japan; ^7^ Department of Medical Oncology, Kanazawa University Cancer Research Institute Kanazawa University Japan

**Keywords:** CTPS, epithelial‐to‐mesenchymal transition, taurine metabolism, TGF‐β

## Abstract

Epithelial‐to‐mesenchymal transition (EMT) contributes to the poor prognosis of patients with cancer by promoting distant metastasis and anti‐cancer drug resistance. Several distinct metabolic alterations have been identified as key EMT phenotypes. In the present study, we further characterize the role of transforming growth factor‐β (TGF‐β)‐induced EMT in non‐small‐cell lung cancer. Our study revealed that TGF‐β plays a role in EMT functions by upregulation of cytidine 5′‐triphosphate synthetase 1 (CTPS), a vital enzyme for CTP biosynthesis in the pyrimidine metabolic pathway. Both knockdown and enzymatic inhibition of CTPS reduced TGF‐β‐induced changes in EMT marker expression, chemoresistance and migration *in vitro*. Moreover, *CTPS* knockdown counteracted the TGF‐β‐mediated downregulation of UDP‐glucuronate, glutarate, creatine, taurine and nicotinamide. These findings indicate that CTPS plays a multifaceted role in EMT metabolism, which is crucial for the malignant transformation of cancer through EMT, and underline its potential as a promising therapeutic target for preventing drug resistance and metastasis in non‐small‐cell lung cancer.

## Graphical abstract

Epithelial‐to‐mesenchymal transition (EMT) contributes to cancer metastasis and drug resistance. Cytidine 5′‐triphosphate synthetase 1 (CTPS) is upregulated by transforming growth factor‐β (TGF‐β), promoting EMT in non‐small‐cell lung cancer. Inhibiting CTPS reduces EMT marker expression, chemoresistance and cell migration. In addition, *CTPS* knockdown reverses TGF‐β‐induced metabolic changes. Therefore, CTPS significantly influences EMT‐related functions and metabolic alterations, highlighting its role in cancer progression.

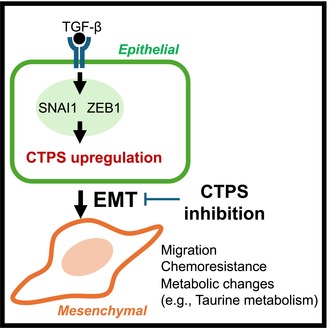

**Abbreviations**
ANOVA: analysis of variance
CDH1: E‐cadherin
CHH2: N‐cadherin
CE‐TOF/MS: capillary electrophoresis time‐of‐flight mass spectrometry
CRC: colorectal cancer
CTPS: cytidine 5′‐triphosphate synthetase 1
DON: 6‐diazo‐5‐oxo‐l‐norleucine
EPCAM: epithelial cell adhesion molecule
EMT: epithelial‐to‐mesenchymal transition
GEO: Gene Expression Omnibus
HIF: hypoxia‐inducible factor
MMP: matrix metalloproteinase
MTT: 3‐(4,5‐dimethylthiazol‐2‐yl)‐2,5‐diphenyltetrazolium bromide
NSCLC: non‐small‐cell lung cancer
PBS: phosphate‐buffered saline
siRNA: small interfering RNA
SNAI1: Snail family transcriptional repressor 1
TCGA: The Cancer Genome Atlas
TGF‐β: transforming growth factor‐β
TWIST1: twist family BHLH transcription factor 1
VIM: vimentin
ZEB1: zinc finger E‐box‐binding homeobox 1

Epithelial‐to‐mesenchymal transition (EMT) is a biological process by which epithelial cells transform into mesenchymal cells. Epithelial cells serve as barriers, preserving the structural integrity of tissues and organs. These properties are vital for cancer cells to invade adjacent tissues, intravasate into the blood or lymphatic vessels, migrate to distant sites and establish secondary tumors. Moreover, EMT can foster the development of cancer stem cells, which possess the capacity to self‐renew and differentiate, thus contributing to tumor heterogeneity and therapeutic resistance [1, 2, 3]. For targeted drugs to disrupt this process and enhance patient outcomes, understanding the processes that propel EMT in cancer is essential.

Transforming growth factor β (TGF‐β) is a multifunctional cytokine with pleiotropic effects on various cellular processes, including cell proliferation, differentiation, apoptosis, cellular senescence and immune regulation [4]. TGF‐β is a potent EMT inducer, downregulating epithelial markers, including E‐cadherin (CDH1) and epithelial cell adhesion molecule (EPCAM) and upregulating mesenchymal markers such as N‐cadherin (CDH2) and vimentin (VIM) [5]. Through SMAD signaling, TGF‐β promotes the expression of various EMT‐inducing transcription factors, including Snail family transcriptional repressor 1 (SNAI1), zinc finger E‐box binding homeobox 1 (ZEB1) and Twist family BHLH transcription factor 1 (TWIST1) [5]. Extensive studies described that these transcription factors are central to EMT and contribute to tumor progression [4, 5].

Metabolic reprogramming is a primary characteristic of cancers. Various distinct metabolic modifications are crucial for cancer cells to attain malignant phenotypes, such as aberrant and excessive proliferation, elevated metastatic potential, and survival under nutrient‐deficient and hypoxic conditions. The majority of proliferating cancer cells activate glycolysis and lactate production over oxidative phosphorylation in the mitochondria for ATP production, even under aerobic conditions. This phenomenon, known as the Warburg effect, supports increased rates of proliferation and tumor growth [6, 7]. Furthermore, studies have shown that changes in nucleotide, lipid and amino acid metabolism in cancer cells are vital for energy provision, biosynthetic resources and oxidative stress tolerance, comprising factors that are indispensable for cancer progression [8, 9].

During EMT, several metabolic pathways, including glycolysis, the tricarboxylic acid cycle, lipid metabolism, and amino acid metabolism, undergo significant changes, correlating with the mesenchymal status of cancer cells [10, 11, 12]. Two of the three subtypes of hexokinase (HK), an enzyme that phosphorylates hexoses like glucose and fructose, are associated with EMT in various cancers. HK2 is upregulated under hypoxic conditions in tongue squamous cell carcinoma and augments migration and invasiveness via EMT [13]. HK3 expression correlates strongly with the expression of EMT‐associated genes such as *ZEB2*, *SNAI2*, *TWIST1* and collagen type I alpha 1 chain (*COL1A1*) in human colorectal cancer specimens [14]. Reduced activity of SDHC, a subunit of succinate dehydrogenase, in hepatocellular carcinoma leads to altered expression of EMT‐related genes, promoting cell migration and invasiveness [15, 16]. Acetyl‐CoA carboxylase, a key enzyme in lipogenesis, is inhibited downstream of TGF‐β in breast cancer cells, resulting in increased acetyl‐CoA levels and induction of EMT via Smad2 acetylation [17]. These insights suggest that the metabolic alterations linked with EMT are essential for upholding the mesenchymal state of cancer cells.

Previously, we have performed a comprehensive metabolic analysis during TGF‐β‐induced EMT in non‐small‐cell lung cancer (NSCLC) and identified specific shifts in amino acid metabolism that contribute to EMT migration and metastasis [18]. In addition, our previous metabolic analysis indicated that TGF‐β significantly alters pyrimidine metabolism; however, its biological role and function remain unknown. In the present study, we found that TGF‐β induced the expression of cytidine 5′‐triphosphate synthetase 1 (CTPS), a key enzyme for CTP biosynthesis in the pyrimidine metabolic pathway, which contributes to EMT progression, anti‐cancer drug resistance and cell migration in NSCLC.

## Materials and methods

### Reagents and cell culture

Recombinant human TGF‐β was acquired from R&D Systems Inc. (Minneapolis, MN, USA). The lung cancer cell lines A549 (CCL‐185), HCC827 (CRL‐2868), H358 (CRL‐5807), H1573 (CRL‐5877), H460 (HTB‐177) and SW1573 (CRL‐2170) were sourced from the American Type Culture Collection (ATCC; Manassas, VA, USA). RERF‐LC‐AD2 (JCRB1021) and LU‐99 (JCRB0080) cells were obtained from the Japanese Cell Research Bank (JCRB; Osaka, Japan).

All cells were maintained in RPMI 1640 medium (FUJIFILM Wako Pure Chemical Industries, Osaka, Japan) enriched with 10% (v/v) fetal bovine serum, 100 U·mL^−1^ penicillin, 100 mg·mL^−1^ streptomycin and 0.25 mg·mL^−1^ amphotericin B. Cultures were incubated at 37 °C in a humidified environment containing 5% CO_2_. The CTPS inhibitor 6‐diazo‐5‐oxo‐l‐norleucine (DON) was procured from Sigma‐Aldrich (St Louis, MO, USA).

### Real‐time PCR analysis

The real‐time PCR was carried out as described previously [18, 19]. Briefly, RNA was isolated from cells using the TRIzol reagent (Thermo Fisher Scientific, Waltham, MA, USA) in accordance with the manufacturer's instructions. Approximately 500 ng of RNA was reverse transcribed employing the First Strand cDNA Synthesis kit (ReverTra Ace α; Toyobo Co., Ltd, Osaka, Japan). The quantitative real‐time PCR was conducted using SYBR premix Ex Taq (Takara, Shiga, Japan) on a StepOnePlus Real‐Time PCR system (Applied Biosystems, Foster City, CA, USA) in accordance with the manufacturer's instructions.

For quantification, the $2^{-\Delta \Delta C_{t}}$ method was utilized, setting ribosomal protein L27 (*RPL27*) expression as an internal standard. Melt‐curve analyses verified the production of all the real‐time PCR products as singular DNA duplexes. All experiments were carried out in triplicate. The primers deployed for real‐time PCR are detailed in Table S1.

### Western blotting

Western blotting was performed as previously described [18]. Proteins were extracted from cell lines utilizing RIPA buffer (Sigma‐Aldrich) or M‐PER reagent (Thermo Fisher Scientific) that contained a protease inhibitor cocktail (Roche Diagnostics, Indianapolis, IN, USA). Protein concentrations were determined using the Bradford protein assay. Equal protein amounts were loaded into each well and separated on 4–15% Mini‐PROTEAN TGX Precast Gels (Bio‐Rad Laboratories, Inc., Hercules, CA, USA) at 100 V for 70 min. These proteins were then transferred onto a poly (vinylidene difluoride) membrane (Bio‐Rad Laboratories, Inc.) utilizing the Trans‐Blot® Turbo™ Transfer System. The membrane was blocked for 3 h with the Blocking One reagent (Nacalai Tesque, Inc., Kyoto, Japan) under agitation at room temperature. Subsequently, the membrane was incubated with a primary antibody diluted in Can Get Signal® Immunoreaction Enhancer Solution 1 (Toyobo Co., Ltd) overnight at 4 °C. Afterward, the membrane was washed thrice in phosphate‐buffered saline (PBS) containing 0.1% Tween 20 and then incubated with a secondary antibody at a 1 : 5000 dilution for 1 h, with agitation at 20 °C. The membrane was again washed three times, each for 5 min. Results were visualized using ECL western blot detection reagents (GE Healthcare Bio‐Sciences, Piscataway, NJ, USA) and captured with a Luminescent Image Analyzer (LAS‐4000 mini; FUJIFILM, Tokyo, Japan). Primary antibodies used included ACTIN (dilution 1 : 5000; sc‐47778; Santa Cruz Biotechnology, Inc., Santa Cruz, CA, USA), CTPS (1 : 1000, 15914‐1‐AP; Proteintech), CDH1 (dilution 1 : 1000; #3195; Cell Signaling Technology, Danvers, MA, USA), CDH2 (dilution 1 : 1000; #14215; Cell Signaling Technology) and GAPDH (dilution 1 : 2000; 10494‐1‐AP; Proteintech, Rosemont, IL, USA). Horseradish peroxidase‐conjugated secondary antibodies (GE Healthcare Bio‐Sciences) were used.

### Metabolite quantification in cells using capillary electrophoresis time‐of‐flight mass spectrometry (CE‐TOF/MS)

Intracellular metabolites were quantified using CE‐TOF/MS (Agilent Technologies, Palo Alto, CA, USA) as described in previous studies [18, 19, 20, 21]. Briefly, cells were rinsed twice with 5% (w/v) mannitol (FUJIFILM Wako Pure Chemical Industries) and lysed in 600 μL of methanol containing internal standards (25 μm each of methionine sulfone, ethane sulfonic acid and d‐camphor‐10‐sulfonic acid). The resulting homogenate was combined with 200 μL of Milli‐Q water (MilliporeSigma, Burlington, MA, USA) and 400 μL of chloroform. Following centrifugation, the isolated methanol–water phase was passed through a 5‐kDa cutoff filter (Millipore, Bedford, MA, USA) to eliminate proteins. The filtrate was lyophilized and then reconstituted in 25 μL of Milli‐Q water with internal standards (200 μm each of 1,3,5‐benzenetricarboxylic acid and 3‐aminopyrrolidine) for analysis via CE‐TOF/MS. The raw data were processed with MasterHands [22]. Metabolite identifications were determined by aligning their *m/z* values and migration times with those of reference compounds.

We performed the principal component analysis (PCA) using the ‘prcomp’ function in the ‘stats’ R package to interpret the data. The metabolites, the concentrations of which were influenced by TGF‐β were analyzed for metabolite set enrichment analysis using metaboanalyst 5.0 (http://www.metaboanalyst.ca) and the Small Molecule Pathway Database library (https://www.smpdb.ca).

### RNA interference

RNA interference was conducted following the method outlined in a previous study [18]. *CTPS* and negative control small interfering RNA (siRNA) duplexes were sourced from Sigma‐Aldrich (negative control, SIC‐001; *CTPS*#1, SASI_Hs02_00332780; *CTPS*#2, SASI_Hs01_00011529; *SNAI1*#1, SASI_Hs01_00039789; *SNAI1*#2, SASI_Hs01_00039791; *TWIST1*#1, SASI_Hs01_00048454; *TWIST1*#2, SASI_Hs01_00048457; *ZEB1*#1, SASI_Hs02_00330529; and *ZEB1*#2, SASI_Hs02_00330530). Cells were plated in six‐well plates and allowed to adhere overnight. They were then transfected with 100 pmol of siRNA oligomer combined with Lipofectamine RNAiMAX reagent (Thermo Fisher Scientific) in serum‐reduced Opti‐MEM (Thermo Fisher Scientific) in accordance with the manufacturer's instructions.

### Immunofluorescence

Cells were cultured in 48‐well plates and fixed with 4% paraformaldehyde for 15 min at room temperature. After fixation, they were washed three times with PBS. Samples were then blocked using PBS Tween‐20 buffer (containing 0.2% Tween‐20 and 0.5% goat serum) and incubated with primary antibodies CDH2 (13A9 mAb; dilution 1 : 1000, #14215; Cell Signaling Technology) overnight at 4 °C. Subsequently, the cells were rinsed with PBS and incubated with Alexa Fluor® 488‐conjugated secondary antibodies (Life Technologies, Gaithersburg, MD, USA) in the dark at room temperature for 2 h. Nuclei were stained using Hoechst 33258 for 15 min. After three subsequent washes with PBS, cells were visualized using the Cytell Cell Imaging System (GE Healthcare UK Ltd, Little Chalfont, UK). For each sample, nine images were captured and analyzed using cytell software (GE Healthcare UK Ltd).

### Cell viability assay

Cell viability was assessed using the 3‐(4,5‐dimethylthiazol‐2‐yl)‐2,5‐diphenyltetrazolium bromide (MTT; Sigma‐Aldrich) assay, conducted as previously described [19, 23]. Briefly, cells (2.5 × 10^3^ cells per well) were seeded into each well of a 96‐well plate and allowed to incubate for 24 h, followed by treatment with erlotinib for another 72 h. Subsequently, 50 μL of MTT solution (2 mg·mL^−1^ in PBS) was introduced to each well and the plates were incubated for 2 h. The formed formazan crystals were solubilized in 100 μL of dimethyl sulfoxide after removing the culture medium. Plates were then agitated on a plate shaker for 1 min and promptly read at a wavelength of 570 nm using a TECAN microplate reader equipped with magellan software (Tecan Group Ltd., Männedorf, Switzerland).

For the trypan blue exclusion assay, 1.5 × 10^5^ cells per well were seeded in 12‐well cell culture plates and incubated at 37 °C for 24 h. Forty‐eight hours after treatment of A549 cells with *CTPS* siRNA, cells were disaggregated in 500 μL of medium and 10 μL of the suspension was mixed with 10 μL of trypan blue (Thermo Fisher Scientific). Viable cells were counted using a Countess Automated Cell Counter (Thermo Fisher Scientific).

### *In vitro* cell migration assays

Transwell migration assays were conducted as described previously [18]. For these assays, approximately 5.0 × 10^4^ cells in 100 μL of serum‐free RPMI 1640 were seeded onto filter inserts (Transwell culture inserts; Corning Costar, Cambridge, MA, USA). The lower chamber was filled with a medium containing 10% fetal bovine serum to serve as a chemoattractant, stimulating cell migration. After an incubation of 4 h, the inserts were fixed and stained using a Diff‐Quik kit (Sysmex Corporation, Hyogo, Japan). Non‐migratory cells were wiped away with cotton swabs and the membranes were air‐dried. The membranes were then photographed using an EVOS microscope (Thermo Fisher Scientific). Three random views for each membrane were inspected to count the cells. Every experiment was performed independently three times. Results are depicted as the number of cells that migrated per field.

### The Cancer Genome Atlas (TCGA) data

Data pertaining to *CTPS* mRNA expression and TNM staging from TCGA Lung Cancer (LUNG) cohort were retrieved from UCSC Xena (https://xena.ucsc.edu). Following the Kruskal–Wallis test, Dunn's multiple comparisons test was conducted for statistical analysis using the prism (GraphPad Software Inc., San Diego, CA, USA).

### Kaplan–Meier survival analysis

Differences in survival based on gene expression were verified using Kaplan–Meier‐Plotter (http://www.kmplot.com/analysis/index.php?p=service&cancer=lung) [24]. ‘*CTPS* (202613_at)’ was input as the gene symbol, and the auto checkbox was selected to determine the optimal cutoff value for *CTPS* mRNA expression, segmenting patients into high‐ and low‐expression categories. Univariate Cox regression analysis was carried out to compute the hazard ratio (accompanied by a 95% confidence interval) and *P*‐values. Two Gene Expression Omnibus (GEO) datasets (GSE30219 and GSE37745) were utilized to assess the relationship between the expression of individual metabolic genes and patient outcomes.

### Microarray analysis

To analyze the gene expression involved in the pyrimidine metabolic pathway, we examined microarray data of the A549 cells treated with or without TGF‐β. Microarray analyses were performed previously (GEO accession: GSE136780) [18].

### Correlation analysis between *CDH1*, *EPCAM*, *VIM* and *CTPS* mRNA expression in lung cancer cell lines

Transcriptome data for 130 NSCLC cell lines were obtained from the CellMinerCDB (https://discover.nci.nih.gov/rsconnect/cellminercdb) [25].

These data were visualized in scatter plots. The regression line is indicated by the blue line. Pearson's correlations and *P*‐values were calculated using R (R Foundation, Vienna, Austria).

### Statistical analysis

All experiments were conducted a minimum of two times, yielding consistent results. Statistical evaluations were undertaken using Excel (Microsoft, Redmond, WA, USA) and r software [26]. For *in vitro* experiments, data from two groups were analyzed using Student's *t*‐test and, for those from more than two groups, appropriate statistical tests were employed. Data are presented as the mean ± SD. *P* < 0.05 was considered statistically significant.

## Results

### TGF‐β‐induced EMT elevates CTPS expression through the canonical transcriptional program of EMT

**Fig. 1:**
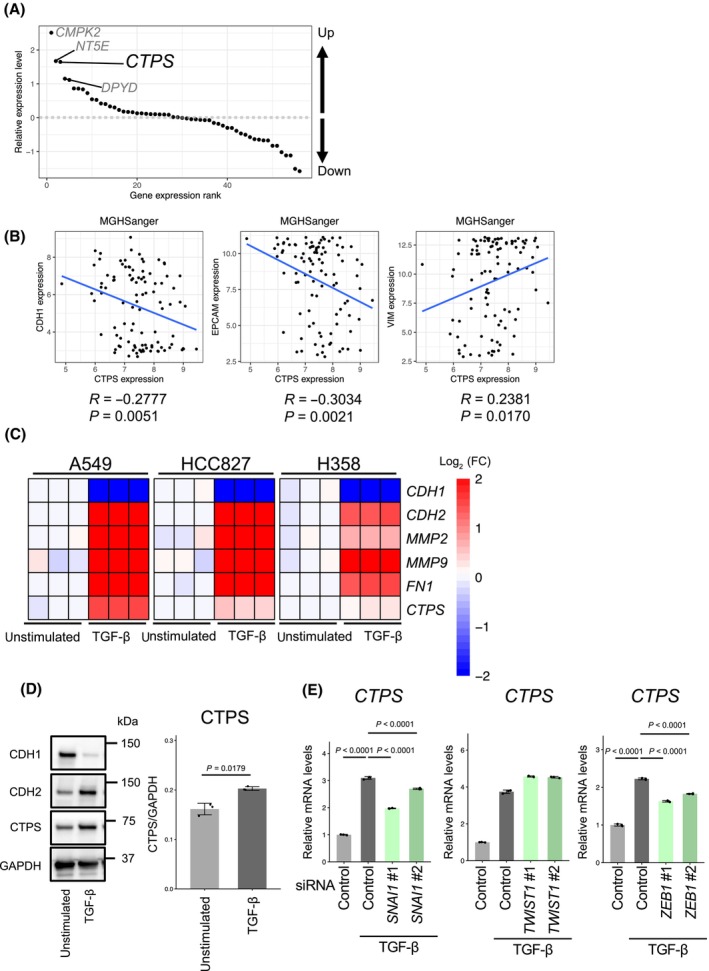
TGF‐β‐induced EMT elevates *CTPS* expression via ZEB1 and SNAI1. (A) Ranking of genes expressed in A549 stimulated with TGF‐β compared to unstimulated cells. (B) Correlations between *CDH1*, *EPCAM*, *VIM* and *CTPS* mRNA expression in NSCLC cell lines in the MGH‐Sanger dataset. (C) Real‐time quantitative PCR analyses of EMT markers (*CDH1*, *CDH2*, *FN1*, *MMP2* and *MMP9*) and *CTPS* in A549, HCC827 and H358 cells following stimulation with TGF‐β (5 ng·mL^−1^) for 2 weeks. Red and blue denote higher and lower mRNA expression levels, respectively than those in unstimulated cells. (D) Protein levels of the EMT markers (CDH1 and CDH2) and CTPS in A549 cells post‐stimulation with TGF‐β (5 ng·mL^−1^) for 72 h (labeled as TGF‐β). GAPDH was used as a loading control for the western blot analysis. (E) Real‐time quantitative PCR analyses of *CTPS* expression. A549 cells transfected with control, *SNAI1*, *TWIST1* or *ZEB1* targeted siRNAs were exposed to 5 ng·mL^−1^ TGF‐β for 48 h. Data are shown as the mean ± SD (*n* = 3). *P* values were determined using one‐way analysis of variance (ANOVA) followed by Dunnett's test.

TGF‐β is known to be one of the most potent EMT inducers. We have previously performed a metabolome analysis of three TGF‐β‐treated NSCLC cell lines and identified the alteration of pyrimidine metabolism (e.g. CTP/UTP metabolism) across all cell lines [18]. To investigate the molecular mechanisms, we used microarray analysis to analyze the gene expression involved in the pyrimidine metabolic pathway and found that *CTPS* expression was augmented during TGF‐β‐induced EMT in A549 cells. The top four pyrimidine‐related metabolic enzyme genes for which expression was increased by TGF‐β were *CMPK2*, *CTPS*, *NT5E* and *DPYD* (Fig. 1A; Table S2). NT5E (i.e. ecto‐5′‐nucleotidase) is a mesenchymal stem cell marker [27] and DPYD (i.e. dihydropyrimidine dehydrogenase) is highly expressed upon EMT induction and is required for cells to acquire mesenchymal characteristics [28]. Given that *CMPK2* and *CTPS* might also have a role in the EMT phenotypes, we analyzed their correlation with EMT status by using the public gene expression databases, Cancer Cell Line Encyclopedia [29, 30] and Sanger/Massachusetts General Hospital GDSC [31, 32, 33], which encompass gene expression of 130 NSCLC cell lines. *CTPS* expression tended to correlate negatively with the expression of the epithelial cell marker genes *CDH1* and *EPCAM* and positively with the mesenchymal marker gene *VIM* but not with *CMPK2* (Fig. 1B; Fig. S1A,B).

We verified the influence of TGF‐β on EMT induction and *CTPS* expression in NSCLC cell lines A549, H827 and H358 in quantitative PCR and western blot analyses (Fig. 1C,D; Fig. S1C,D). The results showed that TGF‐β led to a decrease in *CDH1* expression (epithelial cell marker), whereas expression of mesenchymal cell markers, *CDH2* and fibronectin 1 (*FN1*), and matrix metalloproteinases (MMPs) involved in cell invasion and migration, *MMP‐9* and *MMP‐2*, was elevated. TGF‐β also upregulated *CTPS* expression across three NSCLC cell lines (Fig. 1C,D; Fig. S1C,D).

To delve deeper into the regulatory mechanism underlying *CTPS* expression, we performed siRNA‐mediated knockdown of *SNAI1*, *ZEB1* and *TWIST1* (Fig. S1E), which are recognized EMT‐related transcription factors [34]. Our data indicated that knockdown of both *SNAI1* and *ZEB1* curtailed *CTPS* expression (Fig. 1E), implying that *CTPS* expression is governed by the downstream canonical pathway for EMT induction.

### Knockdown of CTPS suppresses EMT in NSCLC cell lines

**Fig. 2:**
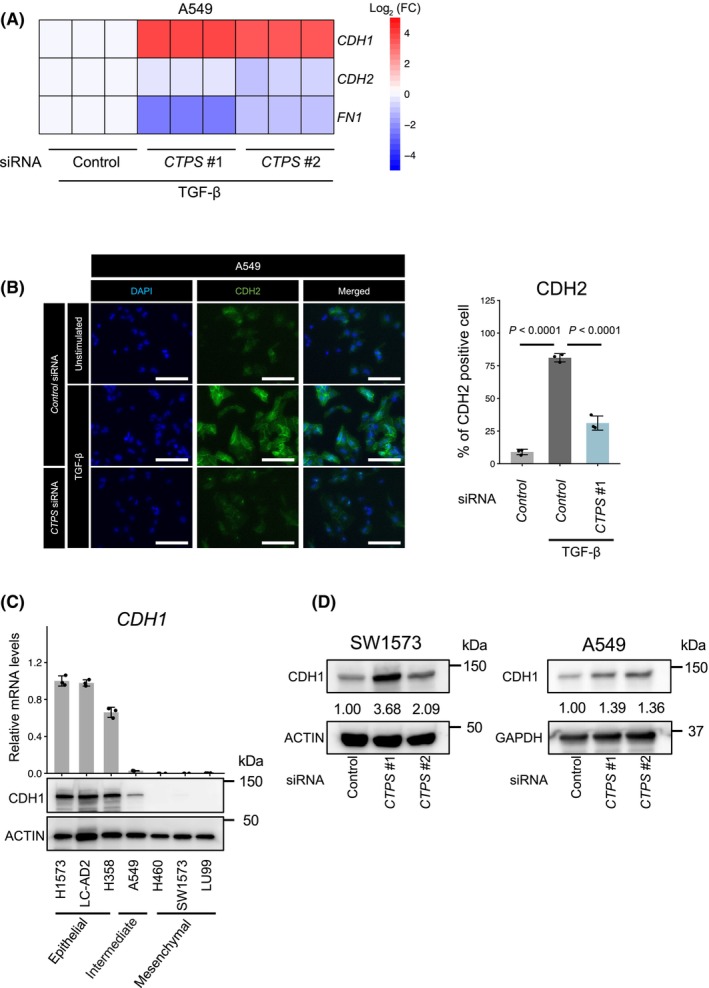
Impact of *CTPS* knockdown on EMT marker expression in NSCLC. (A) Influence of *CTPS* siRNA on EMT marker expression in TGF‐β‐treated A549 cells. Cells transfected with *CTPS* siRNAs were exposed to TGF‐β for 48 h. mRNA expression levels of *CDH1*, *CDH2* and *FN1* were assessed by real‐time PCR. Red and blue denote higher and lower mRNA levels, respectively than those in cells transfected with control siRNA (white). (B) Impact of *CTPS* siRNA on CDH2 protein expression in TGF‐β‐treated A549 cells. Cells transfected with *CTPS* siRNAs were treated with 5 ng·mL^−1^ TGF‐β for 48 h. CDH2 protein levels were evaluated using immunofluorescence staining. Scale bar = 200 μm. Data are shown as the mean ± SD (*n* = 3). *P* values were determined using one‐way ANOVA followed by Dunnett's test. (C) The mRNA and protein expression of CDH1 in mesenchymal and epithelial NSCLC cell lines. Data are shown as the mean ± SD (*n* = 3). (D) Effects of *CTPS* siRNA on protein expression of CDH1 in SW1573 and A549 cells. Actin and GAPDH was used as a loading control for the western blot analyses. Relative protein levels of CDH1 were quantified using imagej (NIH, Bethesda, MD, USA).

Previous studies have shown that alterations in the expression of metabolic enzymes are associated with EMT phenotypes in cancers [11, 28, 35]. To understand this further, we examined the effects of siRNA‐mediated *CTPS* knockdown on the expression of EMT markers in TGF‐β‐treated A549, HCC827 and H358 cells (Fig. 2A,B; Fig. S2A). *CTPS* knockdown increased the mRNA expression of *CDH1*, whereas the levels of *CDH2* and *FN1* decreased (Fig. 2A; Fig. S2A,C). Moreover, immunostaining revealed that TGF‐β‐induced CDH2 protein levels were diminished by *CTPS* knockdown (Fig. 2B). *CTPS* knockdown also slightly decreased the growth of A549 cells (Fig. S2B). Delving deeper into the inherent role of *CTPS* in mesenchymal characteristics, we used SW1573 cells, which exhibit mesenchymal traits characterized by a diminished expression of the epithelial marker CDH1 (Fig. 2C). In SW1573 cells with low CDH1 expression (mesenchymal) and A549 cells with moderate CDH1 expression (intermediate), *CTPS* knockdown augmented the protein level of endogenous CDH1 (Fig. 2D). These observations suggest that CTPS plays a crucial role in TGF‐β‐triggered EMT as well as in preserving inherent mesenchymal characteristics.

### Effect of CTPS knockdown on altered EMT metabolism in TGF‐β‐stimulated lung cancer cells

**Fig. 3:**
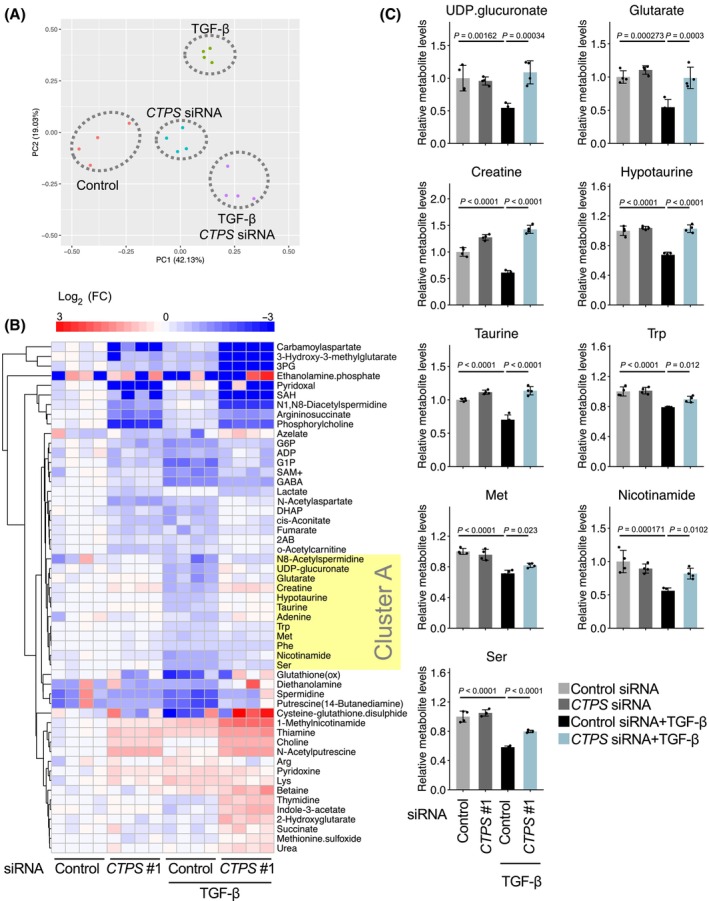
Impact of *CTPS* knockdown on EMT‐induced metabolic changes in TGF‐β‐stimulated NSCLC cells. (A) Principal component analysis of metabolomics profiles from *CTPS*‐knockdown A549 cells, with or without TGF‐β stimulation (*n* = 4). Metabolite levels were determined using CE‐TOF/MS spectrometry. (B) Influence of *CTPS* siRNA on TGF‐β‐induced metabolic alterations in A549 cells. The 53 metabolites that contributed to PC2 (|PC2 coefficient values| > 0.4) (Fig. S3B) are listed. The heat map represents the ratio of the measured sample to the mean concentration of the unstimulated sample. Red and blue signify higher and lower metabolite levels, respectively than those in unstimulated cells (depicted in white). (C) Impact of *CTPS* siRNA on metabolite concentrations. Bar graphs illustrate the metabolite levels compared to those in the control (control siRNA). Data are shown as the mean ± SD from quadruplicate experiments. Data are shown as the mean ± SD (*n* = 4). *P* values were determined using one‐way ANOVA followed by Dunnett's test.

Because CTPS is an important metabolic enzyme for CTP biosynthesis, we investigated its effect on metabolism in TGF‐β‐stimulated A549 cells. Metabolomic analysis using CE‐TOF/MS revealed that *CTPS* knockdown tended to reduce intracellular CTP levels (Fig. S3A) and induced distinctive metabolic alterations, as shown by PCA (Fig. 3A; Table S3). Furthermore, TGF‐β altered the levels of PC2‐associated metabolites, although the changes appeared to be suppressed by *CTPS* knockdown (Fig. 3A). Based on the PC2 coefficients from the PCA, 53 metabolites were identified as contributing to PC2 (|PC2 coefficient values| > 0.4) (Fig. S3B). Hierarchical clustering and heatmap visualization of these metabolites showed that TGF‐β decreased the concentrations of metabolites in cluster A (e.g. UDP‐glucuronate, glutarate, creatine, hypotaurine, taurine, tryptophan, methionine, nicotinamide and serine) (Fig. 3B); conversely, *CTPS* knockdown inhibited these effects (Fig. 3B,C). Metabolite set enrichment analysis for metabolites in cluster A showed that taurine and hypotaurine metabolism is most significant (Fig. S4). These findings suggest that *CTPS* expression induced by TGF‐β regulates specific metabolic pathways such as taurine metabolism.

### Involvement of CTPS in EMT‐associated migratory ability and anti‐cancer drug resistance

**Fig. 4:**
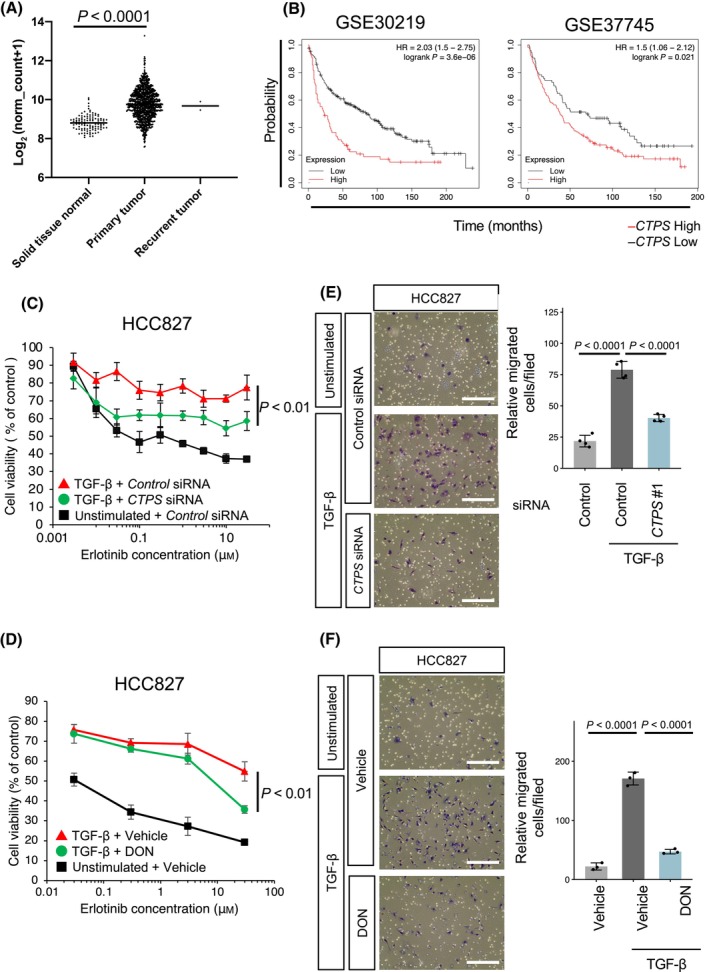
Relationship between *CTPS* expression and lung cancer progression. (A) mRNA expression of *CTPS* in non‐tumor (*n* = 107), primary tumor (*n* = 1008) and recurrent tumor tissues (*n* = 2) sourced from the lung cancer dataset in TCGA. Statistical significance was measured using the Kruskal–Wallis test with Dunn's post‐hoc correction for multiple comparisons. (B) Correlation between *CTPS* expression and prognosis in patients diagnosed with non‐small‐cell lung cancer. (C) Impact of *CTPS* knockdown on erlotinib resistance in TGF‐β‐treated HCC827 cells. HCC827 cells were incubated with 2 ng·mL^−1^ TGF‐β for 2–5 weeks to induce EMT and subsequently transfected with *CTPS* siRNA for 72 h before the cell viability was determined using the MTT assay. Data are shown as the mean ± SD (*n* = 4). Student's *t*‐test was used for statistical analysis. (D) Effect of CTPS inhibitor DON on erlotinib resistance in TGF‐β‐treated HCC827 cells. Cells induced to EMT with TGF‐β were treated with 500 μm DON for 72 h. Data are shown as the mean ± SD (*n* = 4). Student's *t*‐test was used for statistical analysis. (E) Influence of *CTPS* knockdown on migratory activity of TGF‐β‐treated HCC827 cells. HCC827 cells were incubated with 2 ng·mL^−1^ TGF‐β for 2–5 weeks to induce EMT and subsequently transfected with *CTPS* siRNA for 48 h before the cell migration assay. Cells were inspected using bright‐field microscopy at 200× magnification. Scale bar = 250 μm. Data are denoted as the mean ± SD (*n* = 3). *P* values were determined using one‐way ANOVA followed by Dunnett's test. (F) Effect of CTPS inhibitor DON on migratory activity of TGF‐β‐treated HCC827 cells. Cells induced to EMT with TGF‐β were treated with 500 μm DON for 48 h before the cell migration assay. Scale bar = 250 μm. Data are denoted as the mean ± SD (*n* = 3). *P* values were determined using one‐way ANOVA followed by Dunnett's test.

*CTPS* has been documented to play a role in poor prognosis and gemcitabine resistance in pancreatic cancer [36]. We explored the correlation between *CTPS* expression and TNM staging in tumor tissues using the lung cancer dataset from TCGA. Remarkably, *CTPS* expression was elevated in tumor tissues compared to that in non‐tumor tissues (Fig. 4A). Additionally, we analyzed the expression of *CTPS* in clinical samples from two independent studies (GSE30219 and GSE37745) [37, 38] and identified a significant positive correlation (*P* < 0.05) between *CTPS* expression and poor prognosis (Fig. 4B).

TGF‐β‐induced EMT imparts resistance to the anti‐cancer drug erlotinib in HCC827 cells that are otherwise sensitive to this agent [35]. Compared to HCC827 cells cultured under normal conditions, HCC827 cells treated with 2 ng·mL^−1^ TGF‐β for 2–5 weeks displayed increased resistance to erlotinib (Fig. 4C,D), which was notably diminished by siRNA‐mediated *CTPS* knockdown (Fig. 4C). We further evaluated the impact of pharmacological inhibition of CTPS on TGF‐β‐induced erlotinib resistance. The CTPS inhibitor DON significantly mitigated TGF‐β‐induced erlotinib resistance (Fig. 4D), implying that CTPS is instrumental in fostering TGF‐β‐induced erlotinib resistance.

Subsequently, we investigated the functional role of CTPS in NSCLC cell motility by assessing the ramifications of *CTPS* knockdown on the TGF‐β‐induced migratory capacity of HCC827 cells. TGF‐β stimulation enhanced HCC827 cell migration. However, this was markedly curtailed by siRNA targeting *CTPS* (Fig. 4E). Moreover, *CTPS* siRNA suppressed the spontaneous migration ability of the SW1573, A549 and H460 cells with mesenchymal characteristics (Fig. S5). Furthermore, the CTPS inhibitor DON significantly attenuated TGF‐β‐induced migration (Fig. 4F), highlighting the role of CTPS in the EMT‐associated NSCLC migration. These results underpin that CTPS is pivotal in EMT by advancing tumor metastasis and drug resistance through an *in vitro* enzymatic activity‐dependent mechanism.

## Discussion

In the present study, we found that *CTPS* expression was upregulated during TGF‐β‐induced EMT in NSCLC cells. Our data showed that the knockdown of *CTPS* negated the metabolic changes induced by TGF‐β, including UDP‐glucuronate, glutarate, creatine, hypotaurine, taurine, adenine, nicotinamide and several amino acids. Furthermore, the siRNA‐mediated knockdown and pharmacological inhibition of CTPS suppressed EMT‐mediated cell migration and anti‐cancer drug resistance *in vitro*. These findings suggest that CTPS inhibitors are promising candidates for anti‐cancer therapies.

CTPS is pivotal in pyrimidine nucleotide synthesis, catalyzing the conversion of UTP to CTP, comprising an essential component for RNA and DNA synthesis. Earlier research indicates that CTPS is instrumental in the immune system, maintaining the proliferation of activated lymphocytes during immune responses [39]. *CTPS*‐deficient individuals present with combined immune deficiency, typified by recurrent infections from pathogens such as Epstein–Barr virus, Varicella Zoster virus and encapsulated bacteria [40]. Moreover, CTPS has been shown to be associated with cancer aggressiveness. Elevated *CTPS* expression correlates with an adverse prognosis in pancreatic cancer and is more prevalent in gemcitabine‐resistant pancreatic cancer cells [36]. In triple‐negative breast cancer, high *CTPS* expression is linked with a negative prognosis [41].

Previous studies have shown that *CTPS* expression is closely linked with collagen deposition in the pathological tissues of bleomycin‐induced idiopathic pulmonary fibrosis. The EMT process has been reported to be associated with fibrotic lung diseases, including idiopathic pulmonary fibrosis. Additionally, TGF‐β stimulation augmented *CTPS* expression in normal human fibroblasts [42]. These reports align with our data that TGF‐β elevates *CTPS* expression and contributes to the EMT phenotype.

We explored the transcriptional regulatory mechanisms governing EMT‐induced *CTPS* expression. Our findings indicated that TGF‐β‐induced *CTPS* expression was inhibited by the knockdown of *SNAI1* and *ZEB1*, suggesting that *CTPS* expression is regulated downstream of established EMT‐transcription factors. CTPS is reported to be highly expressed in gemcitabine‐resistant pancreatic cancer cell lines, with its expression modulated by hypoxia‐inducible factor (HIF)‐1α [36]. HIF‐1α is known to stabilize downstream of TGF‐β via FAK and Akt activation. Therefore, *CTPS* expression might also be influenced by HIF stabilized through TGF‐β signaling [43]. In addition, our research and that of other groups have revealed that *CTPS* is regulated by *Myc* in colorectal cancer cell lines [44, 45]. Another study also showed that the knockdown of *TWIST*, an EMT‐triggering transcription factor, hinders EMT and *CTPS* expression in gastric cancer cells [46]; however, our results indicated that the knockdown of *TWIST1* did not impact TGF‐β‐induced *CTPS* expression. These studies suggest potential cell type or context specificity in the regulation of *CTPS* expression.

Previous studies demonstrated metabolic shifts during EMT in cancer cells. Our investigation revealed that *CTPS* knockdown partially counteracted the EMT‐induced metabolic alterations. Despite its association with pyrimidine metabolism, *CTPS* knockdown prevented TGF‐β‐induced changes in taurine and hypotaurine levels. Recent studies confirmed that taurine inhibits colorectal cancer (CRC) and triple‐negative breast cancer cell migration and invasion [47, 48]. In CRC, taurine treatment increased *CDH1* and reduced SNAI1 expression, with the latter being a transcription factor linked with EMT [48]. Taurine also suppresses EMT in retinal pigment epithelium differentiated from induced pluripotent stem cells of a patient with mitochondrial myopathy, encephalopathy, lactic acidosis and stroke‐like episodes [49]. In addition, taurine suppressed EMT in retinal pigment epithelium cells and inhibited the phosphoinositide 3‐kinase‐Akt‐mammalian target of rapamycin pathway in triple‐negative breast cancer cell, suggesting its potential as an anti‐metastatic agent [47]. Furthermore, taurine reportedly inhibits A549 cell proliferation both *in vitro* and *in vivo*, indicating its anti‐tumor activity [50]. These results suggest that *CTPS* knockdown might suppress EMT by modulating taurine production. By contrast, hypotaurine, a taurine precursor, reportedly suppresses EMT in CRC and promotes cancer stem‐like cell maintenance in glioma through nuclear factor‐kappa B pathway activation, thereby contributing to cancer progression [48, 51]. In the present study, both taurine and hypotaurine levels were reduced in TGF‐β‐induced EMT and restored upon *CTPS* knockdown. Further investigations would be required to elucidate how CTPS regulates intracellular taurine and hypotaurine levels as well as its involvement in EMT.

The present study highlights the role of CTPS in cancer EMT and suggests it as a promising therapeutic target for preventing drug resistance and metastasis in NSCLC.

## Conflicts of interest

The authors declare that they have no conflicts of interest.

## Author contributions

FN and ST were responsible for study conceptualization. FN was responsible for the formal analysis. FN and ST were responsible for investigations. FN and ST were responsible for the methodology. FN and ST were responsible for data curation. FN was responsible for visualization. FN, AH, TS and ST were responsible for funding acquisition. FN was responsible for writing the original draft. AH, SY and ST were responsible for resources. AH, HM, SY, TS and ST were responsible for supervision, as well as reviewing and editing. ST was responsible for project administration.

## Supporting information


**Fig. S1.** Correlations and expression analyses of EMT markers and *CTPS* in NSCLC cell lines.
**Fig. S2.** Effects of *CTPS* knockdown on EMT marker expression and cell viability in NSCLC cell lines.
**Fig. S3.** Impact of *CTPS* knockdown on CTP levels and metabolic profiling in A549 cells.
**Fig. S4.** Metabolite set enrichment analysis for the metabolites in cluster A.
**Fig. S5.** Influence of *CTPS* knockdown on the migratory activity of H460, A549 and SW1573 cells.

**Table S1.** Primer sequences.
**Table S2.** Expression of genes related to the pyrimidine metabolic pathway in TGF‐β‐treated A549 cells.
**Table S3.** Levels of metabolites in the *CTPS*‐knockdown A549 cells stimulated with or without TGF‐β.

## Data Availability

Metabolome data are included in the Supporting information. Microarray data were deposited in the National Center for Biotechnology Information GEO with the accession code GSE136780. All other data will be available from the corresponding author upon reasonable request.
