# Supplementary material for: The role of cytidine 5′‐triphosphate synthetase 1 in metabolic rewiring during epithelial‐to‐mesenchymal transition in non‐small‐cell lung cancer
Source: FEBS Open Bio. 2024 Jul 19;14(9):1570–83. doi: 10.1002/2211-5463.13860 (PMC11492420; doi:10.1002/2211-5463.13860)
Supplement: Supplementary file 1 — Fig. S1. Correlations and expression analyses of EMT markers and CTPS in NSCLC cell lines. Fig. S2. Effects of CTPS knockdown on EMT marker expression and cell viability in NSCLC cell lines. Fig. S3. Impact of CTPS knockdown on CTP levels and metabolic profiling in A549 cells. Fig. S4. Metabolite set enrichment analysis for the metabolites in cluster A. Fig. S5. Influence of CTPS knockdown on the migratory activity of H460, A549 and SW1573 cells. [file FEB4-14-1570-s001.pdf]

Fig. S1

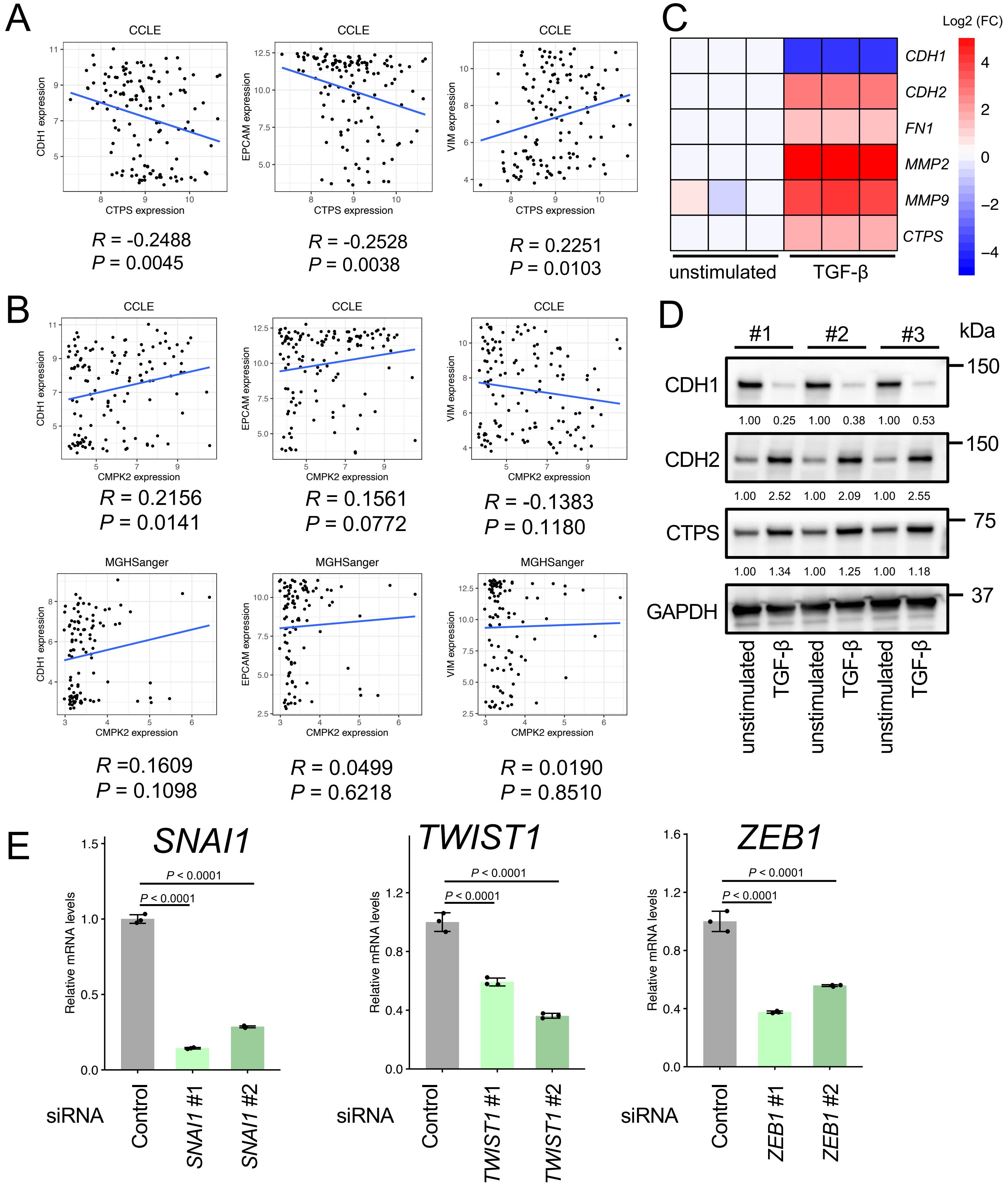

Fig. S1. Correlations and expression analyses of EMT markers and CTPS in NSCLC cell lines. (A) Correlations between *CDH1*, *EPCAM*, *VIM,* and *CTPS* mRNA expression in non-small-cell lung cancer (NSCLC) cell lines in the Cancer Cell Line Encyclopedia (CCLE) datasets. (B) Correlations between *CDH1*, *EPCAM*, *VIM,* and *CMPK2* mRNA expression in NSCLC cell lines in the CCLE and MGHSanger datasets. (C) Real-time quantitative PCR analyses of EMT markers (*CDH1*, *CDH2*, *FN1*, *MMP2*, and *MMP9*) and *CTPS* in A549 cells following stimulation with TGF-β (5 ng/mL) for 72 h. Red and blue denote higher and lower mRNA expression levels, respectively, compared to those in unstimulated cells. (D) Western blot used to quantify CTPS protein levels (n = 3). Relative protein levels were quantified using ImageJ software. (E) Efficiency of *SNAI1*, *TWIST1*, and *ZEB1* siRNA knockdown in A549 cells *in vitro*. mRNA levels in each sample were assessed using real-time PCR. Data are presented as mean ± SD from triplicate samples. *P* values were determined using one-way ANOVA followed by Dunnett's test.

Fig. S2

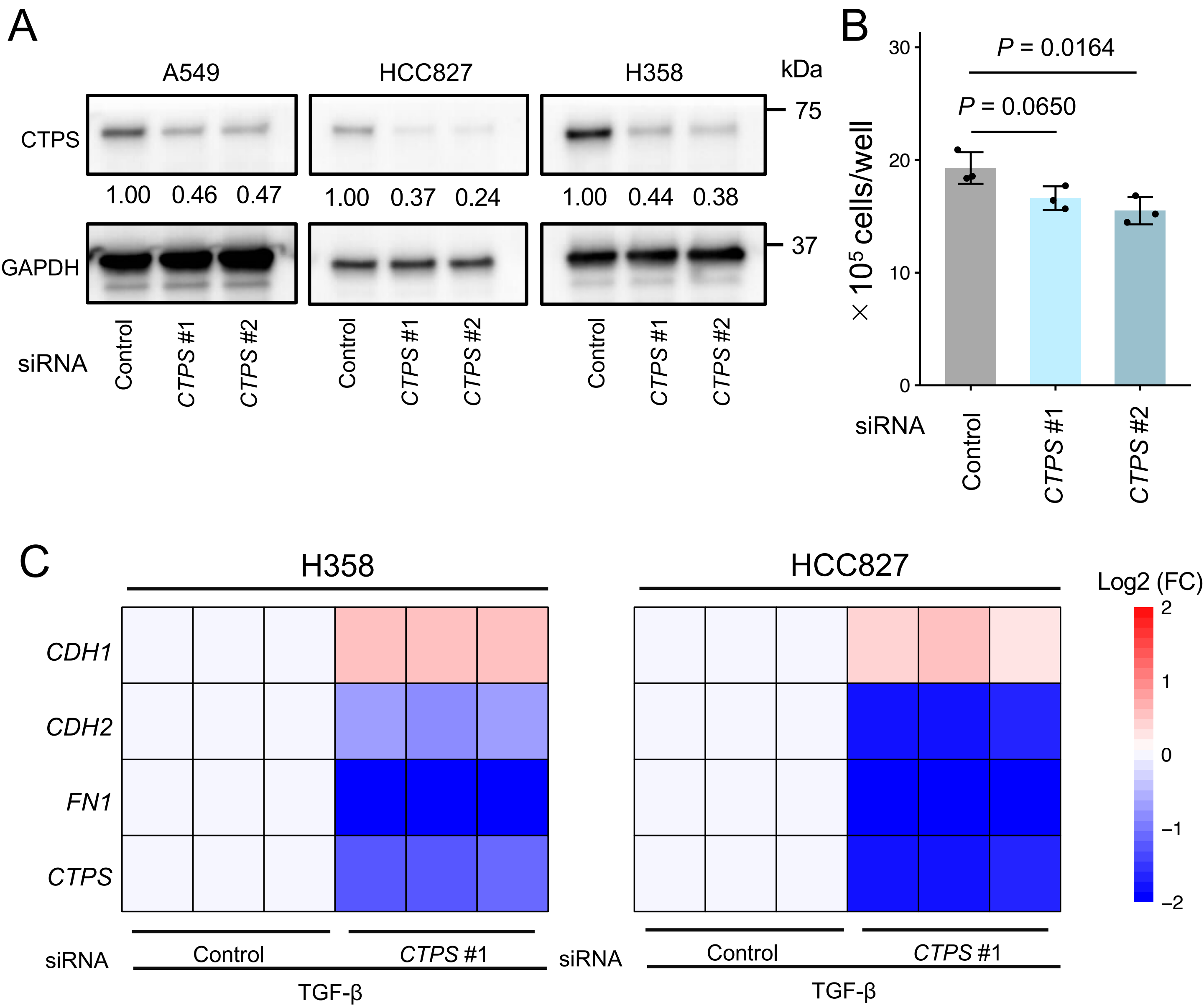

Fig. S2. Effects of CTPS knockdown on EMT marker expression and cell viability in NSCLC cell lines. (A) CTPS expression in A549, HCC827, and H358 cells transfected with *CTPS* siRNA detected by western blotting. Relative protein levels of CTPS were quantified using ImageJ software. (B) Effects of *CTPS* knockdown on cell viability in A549 cells. Data are denoted as mean ± SD (n = 3). *P* values were determined using one-way ANOVA followed by Dunnett's test.(C) Impact of *CTPS* knockdown on mRNA expression levels of EMT marker genes (*CDH1, CDH2*, and *FN1*) and *CTPS* in H358 and HCC827 cells, both stimulated and unstimulated with 5 ng/mL TGF-β. mRNA levels were ascertained using real-time PCR. Data are presented as mean ± SD from triplicate samples.

Fig. S3

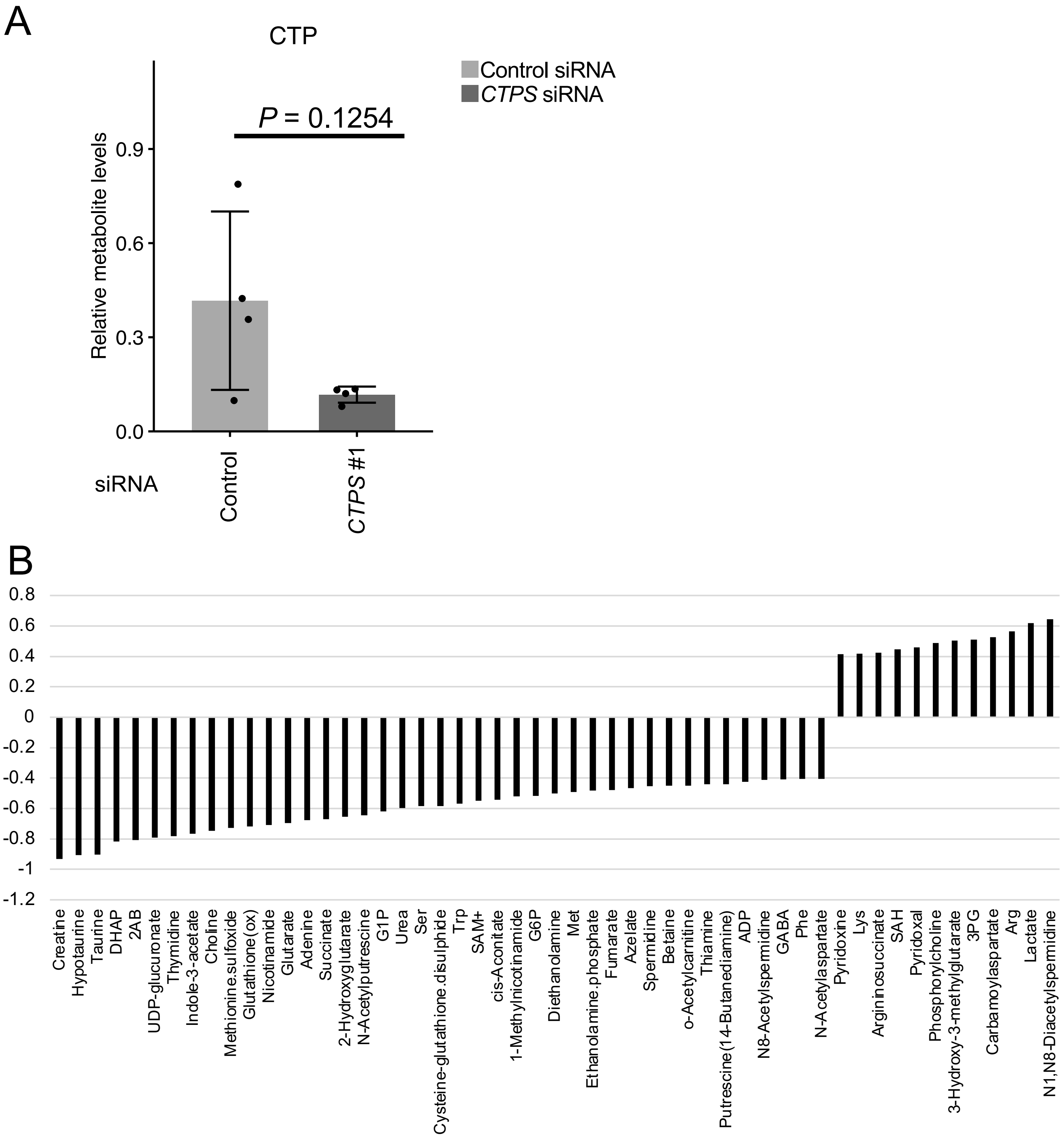

Fig. S3. Impact of CTPS knockdown on CTP levels and metabolic profiling in A549 cells. (A) Effect of *CTPS* knockdown on CTP levels in A549 cells. Data are shown as the mean ± SD (n = 4). Student's t-test was used for statistical analysis. (B) Factor loadings for PC2. Metabolites are organized based on coefficient values. The bar plot highlights metabolites with |PC2 coefficient values| > 0.4 in PCA.

Fig. S4

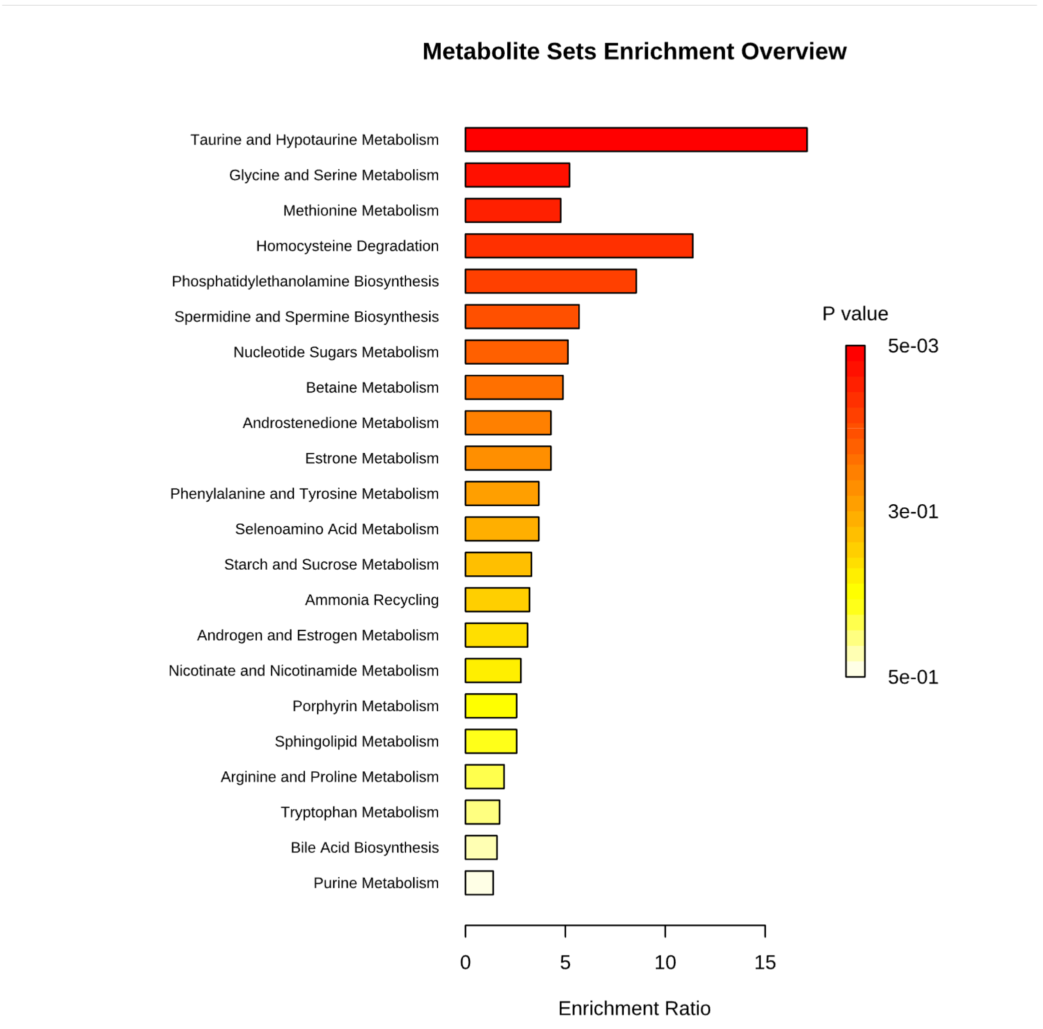

Fig. S4. Metabolite set enrichment analysis for the metabolites in cluster A.

Fig. S5

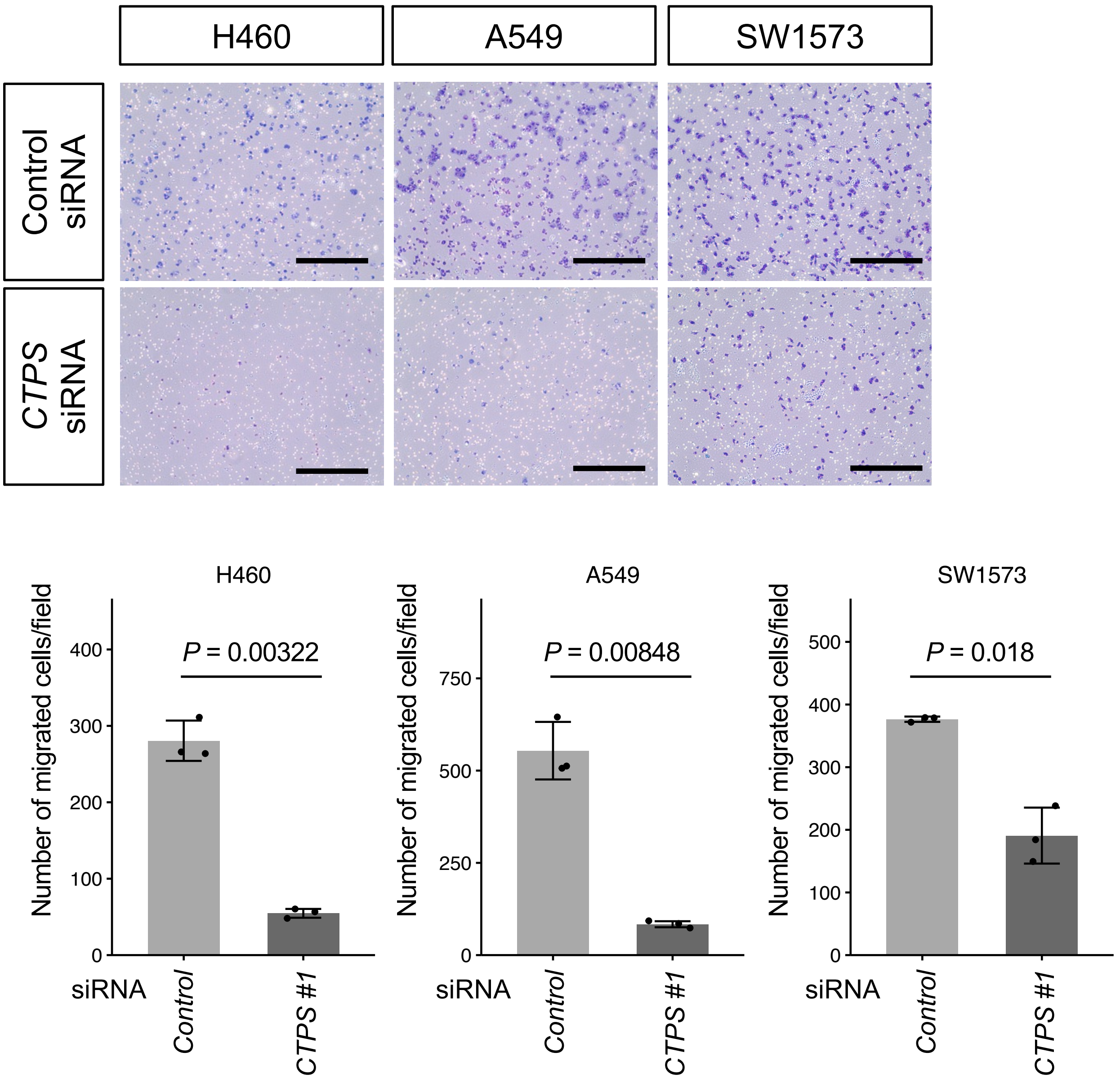

Fig. S5. Influence of *CTPS* knockdown on the migratory activity of H460, A549, and SW1573 cells. Cells were transfected with *CTPS* siRNA for 48 h before the cell migration assay. Cells were inspected using bright-field microscopy at ×100 magnification. Scale bar, 500 µm. Data are denoted as mean ± SD (n = 3). Student's t-test was used for statistical analysis.
